# Identification of a WNT5A-Responsive Degradation Domain in the Kinesin Superfamily Protein KIF26B

**DOI:** 10.3390/genes9040196

**Published:** 2018-04-05

**Authors:** Edith P. Karuna, Shannon S. Choi, Michael K. Scales, Jennie Hum, Michael Cohen, Fernando A. Fierro, Hsin-Yi Henry Ho

**Affiliations:** Department of Cell Biology and Human Anatomy, School of Medicine, University of California, Davis, CA 95616, USA; epkaruna@gmail.com (E.P.K.); shannon.choi.1@gmail.com (S.S.C.); mkscales@umich.edu (M.K.S.); jlhum@ucdavis.edu (J.H.); mdcohen91@gmail.com (M.C.); ffierro@ucdavis.edu (F.A.F.)

**Keywords:** WNT5A, KIF26B, ROR receptors, Frizzled receptors, Dishevelled, GSK3, protein degradation, mesenchymal stem cells

## Abstract

Noncanonical WNT pathways function independently of the β-catenin transcriptional co-activator to regulate diverse morphogenetic and pathogenic processes. Recent studies showed that noncanonical WNTs, such as WNT5A, can signal the degradation of several downstream effectors, thereby modulating these effectors’ cellular activities. The protein domain(s) that mediates the WNT5A-dependent degradation response, however, has not been identified. By coupling protein mutagenesis experiments with a flow cytometry-based degradation reporter assay, we have defined a protein domain in the kinesin superfamily protein KIF26B that is essential for WNT5A-dependent degradation. We found that a human disease-causing KIF26B mutation located at a conserved amino acid within this domain compromises the ability of WNT5A to induce KIF26B degradation. Using pharmacological perturbation, we further uncovered a role of glycogen synthase kinase 3 (GSK3) in WNT5A regulation of KIF26B degradation. Lastly, based on the identification of the WNT5A-responsive domain, we developed a new reporter system that allows for efficient profiling of WNT5A-KIF26B signaling activity in both somatic and stem cells. In conclusion, our study identifies a new protein domain that mediates WNT5A-dependent degradation of KIF26B and provides a new tool for functional characterization of noncanonical WNT5A signaling in cells.

## 1. Introduction

The WNT family of secreted glycoproteins functions as signaling cues in metazoans to orchestrate a diverse array of developmental and regenerative processes [1,2,3]. In the canonical mode of WNT signaling, WNTs promote the stabilization of the downstream effector protein β-catenin, which functions as a transcriptional co-activator to activate target gene expression [2,3]. Moreover, a subset of WNTs are also known to function independently of β-catenin-mediated transcription to regulate cytoskeleton-driven morphogenetic processes, such as cell migration, cell polarization and cell adhesion [1,4]. The molecular mechanisms that underlie this “noncanonical” mode of WNT signaling are more diverse and have continued to remain less well understood. Interestingly, a number of recent studies have shown that noncanonical WNTs can modulate the cellular stability of downstream cytoskeletal effectors via the ubiquitin-proteasome system. For example, WNT5A—the prototypic noncanonical WNT—was previously shown to induce the proteasomal degradation of the cell adhesion molecules syndecan-4 and paxillin [5,6,7]. More recently, WNT5A was found to control the degradation of the kinesin superfamily protein member KIF26B, an evolutionarily conserved modulator of cell adhesion, cell polarization and cell migration [8,9,10]. The biochemical mechanisms by which WNT5A regulates effector degradation, however, are currently unknown. To date, no protein domains that mediate the WNT5A-dependent effector degradation responses have been identified. In this study, we report that the C-terminal end of KIF26B contains a WNT5A-responsive degradation domain that is required for WNT5A-dependent degradation. Importantly, disruption of the domain by a human KIF26B missense mutation recently identified in patients with spinocerebellar ataxia impaired WNT5A-dependent degradation of KIF26B [11]. 

Through pharmacological perturbation experiments, we further identified a role of glycogen synthase kinase 3 (GSK3) in WNT5A regulation of KIF26B degradation. Lastly, using the new knowledge about the WNT5A-regulated degradation domain, we developed a novel live-cell reporter assay for profiling WNT5A-KIF26B signaling activity in both somatic and stem cells. Thus, our study reveals new insights into the biochemical mechanisms by which WNT5A regulates KIF26B stability and adds to the current repertoire of molecular tools for assaying noncanonical WNT signaling activities in cells.

## 2. Materials and Methods 

### 2.1. Cell Lines

The following cell lines were commercially purchased: NIH/3T3 Flp-In (Thermo Fisher Scientific, Hanover Park, IL, USA); HEK293T (ATCC, Manassas, VA, USA); P19 (ATCC). Human mesenchymal stem cells (MSCs) were isolated from purchased fresh bone marrow from healthy donors (StemExpress, Folsom, CA, USA), as described in Reference [12] and used between passages 2 and 5. NIH/3T3 Flp-In and HEK293T cells were cultured in Dulbecco’s Modified Eagles Medium (Corning Inc., Tewksbury, MA, USA) supplemented with 1× glutamine (Corning Inc.), 1× penicillin-streptomycin (Corning Inc.) and 10% fetal bovine serum (Thermo Fisher Scientific). P19 cells were cultured in Alpha Minimum Essential Medium (GE, Pittsburgh, PA, USA) supplemented with 1× penicillin-streptomycin (Corning Inc.), 7.5% bovine calf serum (Thermo Fisher Scientific) and 2.5% fetal bovine serum (Thermo Fisher Scientific). MSCs were cultured in Dulbecco’s Modified Eagles Medium (Corning Inc.) supplemented with 1× glutamine (Corning Inc.), 1× penicillin-streptomycin (Corning Inc.) and 10% fetal bovine serum (Atlanta Biologicals, Lawrenceville, GA, USA). During the expansion phase of MSC cultures, the medium was also supplemented with fibroblast growth factor 2 (FGF-2) (10 ng/mL, Thermo Fisher Scientific). FGF-2 was removed prior to the lentiviral infection, puromycin selection and WNT5A stimulation procedures. All cell lines were cultured at 37 °C and 5% CO_2_.

### 2.2. Protein Mutagenesis 

All KIF26B variants are of mouse origin. The plasmid pEF5-FRT-V5-DEST-GFP-KIF26B ([8]; Addgene, Cambridge, MA, USA) and its derivatives were used as the templates for all molecular biology procedures. Constructs encoding KIF26B truncation variants were generated by PCR using the forward and reverse primers listed in Table A1 (see Appendix A) and inserted into pENTR-2B vectors modified to generate N-terminal or C-terminal green fluorescent protein (GFP) fusion proteins. The ligation reactions were performed using either standard restriction digest/ligation protocols via the FseI and AscI sites or Gibson assembly [13]. The D1904N substitution mutation was introduced into pENTR-2B-GFP-KIF26B-C following the QuikChange Site-Directed Mutagenesis method (Agilent, Santa Clara, CA, USA). All DNA constructs were confirmed by Sanger sequencing.

### 2.3. Generation of Stable NIH/3T3 Lines

The procedure for generating stable NIH/3T3 cells using the Flp-In system was described previously [8,14]. In brief, pENTR-2B plasmids carrying GFP fusions of the various KIF26B mutants were recombined with the pEF5-FRT-V5 vector (Thermo Fisher Scientific) using LR clonase II (Thermo Fisher Scientific). The resulting constructs were transfected into NIH/3T3 Flp-In cells using Genjet (SignaGen Laboratories, Rockville, MD, USA) or X-tremeGene 9 (Roche, Indianapolis, IN, USA) reagents. Stable cell lines were selected using 0.2 mg/mL hygromycin B (Corning Inc.).

### 2.4. Molecular Cloning of KIF26A-C

An EST clone containing a partial mouse *Kif26a* complementary DNA (cDNA) was purchased from GE (GenBank: CD348409.1). DNA encoding KIF26A-C was PCR amplified from this EST clone using primers listed in Table A1 (see Appendix A), subcloned into a modified pENTR-2B-GFP plasmid using the FseI and AscI restriction sites and verified by Sanger sequencing. 

### 2.5. Recombinant Proteins and Inhibitors

The following recombinant protein and inhibitors were purchased: WNT5A (R&D Systems, Minneapolis, MN, USA); Wnt-C59 (Cellagen Technology, San Diego, CA, USA); CHIR99021 (ApexBio, Houston, TX, USA). 

### 2.6. WNT5A Stimulation and Flow Cytometry

Detailed protocols for WNT5A stimulation and the flow cytometry-based GFP-KIF26B degradation assay were described previously [8,14]. Briefly, two days before the WNT5A stimulation experiment, NIH/3T3 reporter cells were plated at a density of 90,000 cells/well in a poly-d-lysine-coated 48-well plate, such that the cell density would be completely confluent on the day of the WNT5A stimulation experiment. P19 cells were plated in a similar fashion in 48-wells to achieve 90–100% confluency on the day of WNT5A stimulation. MSCs were plated in 12-wells and the confluency on the day of WNT5A stimulation was 90–100%. Approximately 24 h before WNT5A stimulation, cells were fed with fresh culture media containing 10 nM Wnt-C59, which inhibits the production of endogenous WNT proteins. Cells were then stimulated with 200 ng/mL WNT5A in Wnt-C59-containing culture media for 6 h. Each WNT5A-stimulated well had a corresponding control well of cells that were mock treated with the control buffer (1× phosphate-buffered saline (PBS), 0.1% w/v bovine serum albumin, 0.5% w/v CHAPS (3-[(3-cholamidopropyl)dimethylammonio]-1-propanesulfonate), diluted at a ratio of 1/200 into Wnt-C59-containing culture media) for 6 h. For treatment with the GSK3 inhibitor, cells were pre-treated for 1 h with CHIR99021 at the indicated concentrations before WNT5A stimulation and the inhibitor was maintained throughout the 6-h WNT5A stimulation period. At the end of WNT5A stimulation, cells were washed once with cold PBS, collected by trypsinization and analyzed by flow cytometry using the BD FACScan system equipped with a 488 nm laser line (Becton Dickinson, San Jose, CA, USA). Cells were kept on ice during the entire period between harvesting and the flow cytometry analysis. For quantification, cells from each experimental condition were plated and treated in triplicate wells. Raw data from the cytometer were acquired using CellQuest (Becton Dickinson) and analyzed in FlowJoX (FlowJo, Ashland, OR, USA). Data processing entailed gating of live cells via side scatter and forward scatter parameters, generating a histogram of GFP fluorescence versus cell count for the live-gated population, overlaying of histograms from compared experimental conditions and calculating the median fluorescence and percent change of median fluorescence between compared conditions. Dose-response analyses were performed using MATLAB with the doseResponse function (written by Ritchie Smith and publicly available on Matlab File Exchange, File ID#33604).

### 2.7. Lentivirus-Mediated Protein Expression

To generate recombinant lentiviruses expressing the GFP-KIF26B-C fusion protein, the pENTR-2B-GFP-KIF26B-C plasmid was recombined with the pLEX_307 lentiviral transfer vector (a gift from David Root; Addgene) using LR clonase II (Thermo Fisher Scientific). Lentiviruses were produced in HEK293T cells using the following third-generation packaging plasmids: pMD2.G, pRSV-rev and pMDLg/pRRE (Addgene) [15]. Viral supernatants collected from HEK293T cultures were used directly to infect target cells without additional purification or concentration steps. Infections were carried out for 16–24 h in the presence of 8 μg/mL polybrene. Following removal of the viruses, cells were allowed to recover for one day in the regular culture media and then selected over 3–5 days using puromycin at the following concentrations: NIH/3T3: 1.5 μg/mL; P19: 0.5 μg/mL; MSCs: 1 μg/mL. Lentiviruses expressing FZD1, SHISA2 and DVL1 were previously described [8].

### 2.8. Protein Sequence Analysis

Protein sequence alignment was performed with ClustalW [16]. Secondary structure prediction was conducted using PSIPRED [17] and COILS [18]. All software programs are publically available online. 

## 3. Results

### 3.1. The C-Terminus of KIF26B Contains a WNT5A-Responsive Degradation Domain

**Figure 1:**
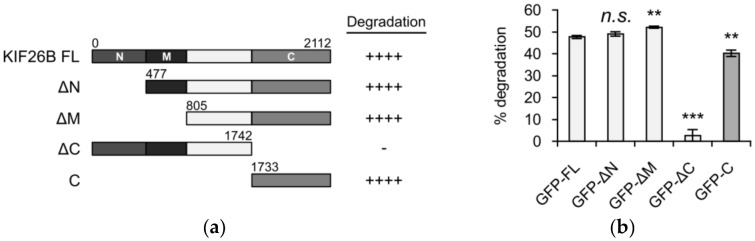

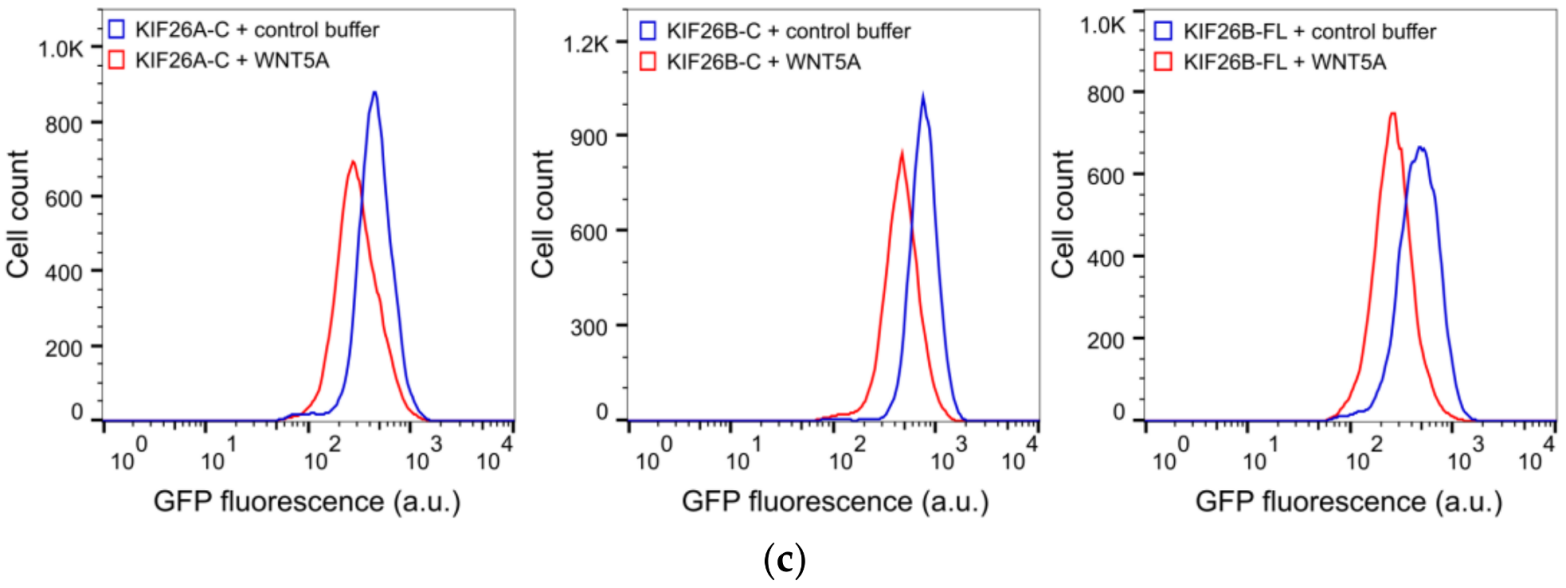
The C-terminus of KIF26B is sufficient and required for WNT5A-dependent degradation. (**a**) Truncation analysis of KIF26B degradation activity in full length KIF26B (FL) and combinations of N-terminal (N), motor-like (M) and C-terminal (C) domains. Each truncated construct was fused to GFP, stably transfected into NIH/3T3 cells and tested for its ability to undergo degradation after WNT5A stimulation (200 ng/mL WNT5A for 6 h) of the reporter cells; (**b**) Quantification of reporter activity for each truncation variant shown in (**a**). Error bars represent ± standard error of the mean (SEM) calculated from three replicates. Statistical analysis was performed with unpaired *t*-test (*n.s.* = not significant; ** *p* value < 0.01; *** *p* value < 0.001 vs. GFP-FL); (**c**) Flow cytometry histograms depicting that GFP-KIF26A-C undergoes WNT5A-dependent degradation with an activity comparable to that of GFP-KIF26B-C and GFP-full-length KIF26B (a.u. = arbitrary units).

KIF26B is a large (~220 kD) protein whose primary protein sequence can be roughly divided into four regions: an N-terminal region followed by a motor-like domain, a central region that contains no known protein domains and a C-terminal region that contains a predicted coiled coil motif. In our previous work, we showed that WNT5A-dependent KIF26B degradation can be measured in a reporter system in which the full-length KIF26B protein is fused to GFP and stably expressed in NIH/3T3 cells, a WNT5A-responsive cell type [8,14]. Upon WNT5A stimulation, the GFP-KIF26B fusion protein undergoes degradation and the decrease in GFP fluorescence can be measured quantitatively by flow cytometry. To determine the region(s) of KIF26B responsible for the degradation response, we generated and tested NIH/3T3 reporter lines that express truncation variants spanning different parts of KIF26B (Figure 1a). As shown in Figure 1b, all truncation variants containing the C-terminal 378 amino acids (KIF26B-C) exhibited robust WNT5A-dependent degradation responses indistinguishable from the full-length GFP-KIF26B reporter. Conversely, the truncation variant lacking KIF26B-C (GFP-KIF26B-ΔC) no longer exhibited any WNT5A-dependent degradation response (Figure 1b). Together, these results indicate that KIF26B-C is sufficient and required for WNT5A-dependent KIF26B degradation.

KIF26B is part of the N-11 kinesin family that also includes KIF26A in vertebrates [19]. Protein sequence comparison showed that the C-terminal region is highly conserved between KIF26A and KIF26B, raising the possibility that the ability of this region to mediate WNT5A-dependent degradation is also conserved. To test this hypothesis, we cloned the mouse KIF26A-C region, expressed it in NIH/3T3 cells and tested its function in the degradation reporter assay. Indeed, KIF26A-C degrades robustly upon WNT5A stimulation, and the extent of degradation is comparable to that of KIF26B-C or full length KIF26B (Figure 1c). We conclude that, like KIF26B, KIF26A is also a regulatory target of the noncanonical WNT5A pathway and that the function of the C region in mediating WNT5A-dependent degradation is conserved across the KIF26 family.

### 3.2. Defining the Sequence Elements within KIF26B-C that are Essential for WNT5A-Dependent Degradation

**Figure 2:**
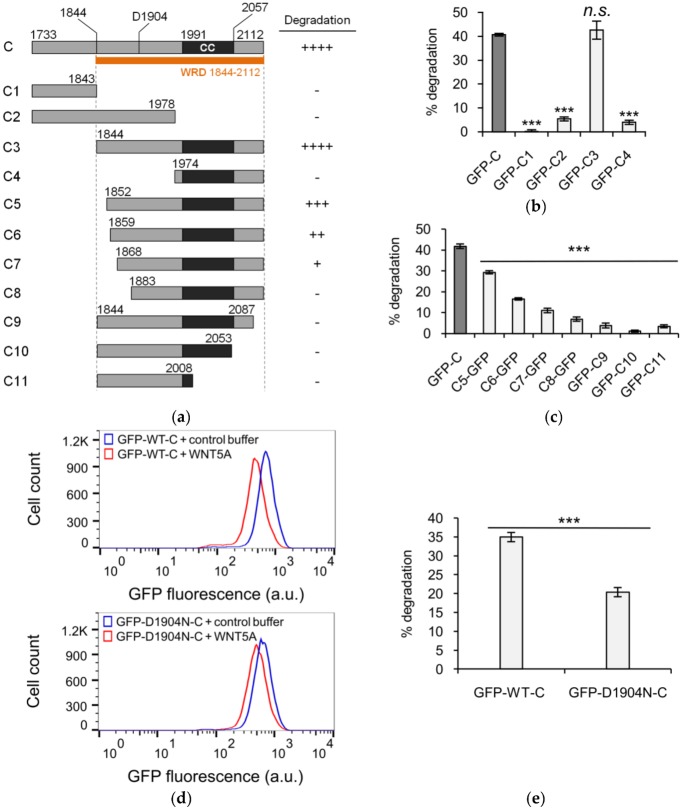
The WNT5A-responsive degradation (WRD) domain of KIF26B is physiologically significant. (**a**) Truncation and mapping analysis of the KIF26B C-terminus. GFP was fused to either the N-terminus (C1–C4, C9–C11) or C-terminus (C5–C8) of each truncation variant and the constructs were analyzed as in Figure 1a. CC, coiled coil; (**b**) Quantification of reporter activity for truncation variants C1–C4 as shown in (**a**). Error bars represent ± SEM calculated from three replicates. Statistical analysis was performed with unpaired *t*-test (*n.s.* = not significant; *** *p* value < 0.001 vs. GFP-C); (**c**) Quantification of reporter activity for fine truncation variants C5–C11 as shown in (**a**). Within this set, GFP was fused to either the N-terminus (C9–C11) or C-terminus (C5–C8) depending on the direction of truncation. We avoided fusing GFP to the end that was being truncated and tested to prevent potential steric hindrance by the GFP tag. Column labels are intended to reflect the position of the GFP tag in each construct. Error bars represent ± SEM calculated from three replicates. Statistical analysis was performed with unpaired *t*-test (*** *p* value < 0.001 vs. GFP-C); (**d**) Flow cytometry histograms depicting the effect of D1904N point mutation on WNT5A-dependent KIF26B-C degradation (a.u. = arbitrary units); (**e**) Quantification of (**d**). Error bars represent ± SEM calculated from independent replicates (GFP-WT-C, *n* = 16; GFP-D1904N-C, *n* = 23). Statistical analysis was performed with unpaired *t*-test (*** *p* value < 0.001 vs. GFP-WT-C).

Having established KIF26B-C as the part of KIF26B that mediates WNT5A-dependent degradation, we next sought to identify the function-related sequence elements within this domain. KIF26B-C contains ~380 amino acids, including the predicted coiled coil motif. To narrow down the relevant sequence element(s), we performed an additional round of more refined truncation analyses (Figure 2a). Successive deletion of amino acids from the N-terminal end of KIF26B-C identified a 39-amino acid stretch (amino acids 1844–1883) preceding the coiled coil motif that is required for WNT5A-dependendent degradation (C3–C8, Figure 2a). Partial deletion within this stretch resulted in partial inhibition of the degradation response, indicating that this 39-amino acid region plays a crucial role for degradation (Figure 2b,c). For the truncation variants beginning with amino acid 1844, deletion from the C-terminal end, either with or without removing the coiled coil, resulted in poorly expressed proteins that do not degrade in response to WNT5A stimulation (Figure 2c). Thus, amino acids 1844–2112 define the minimal sequence required for WNT5A-dependent degradation. We name this sequence element the WNT5A-responsive degradation (WRD) domain. Given that coiled coil motifs are often found to mediate protein dimerization or multimerization and that the individual amino acids within the WRD coiled coil motif are not highly conserved between KIF26A and KIF26B, we favor a model in which the coiled coil motif plays a structural role in maintaining the overall conformation of the WRD domain and that the sequences preceding the coiled coil motif are more directly involved in receiving the WNT5A signal. 

Recently, a human missense mutation (D1904N) in KIF26B was found to cause spinocerebellar ataxia, a progressive neurodegenerative disorder, in a patient cohort [11]. How this mutation affects the function and/or regulation of the KIF26B protein is currently unknown. Interestingly, the D1904N mutation is located within the WRD domain, between the critical 39-amino acid stretch and the coiled coil motif, raising the possibility that the mutation might affect WNT5A-dependent degradation of KIF26B. To test this hypothesis, we introduced the mutation into the GFP-KIF26B-C reporter and assessed the WNT5A-dependent degradation response. We found that the D1904N substitution significantly reduces the ability of KIF26B-C to undergo degradation after WNT5A stimulation (Figure 2d,e). This finding highlights the physiological importance of the WRD domain and further suggests that defects in WNT5A regulation of KIF26B degradation may underlie the etiology of KIF26B-driven neurological disorders.

### 3.3. The Role of GSK3 in WNT5A Regulation of KIF26B Degradation

**Figure 3:**
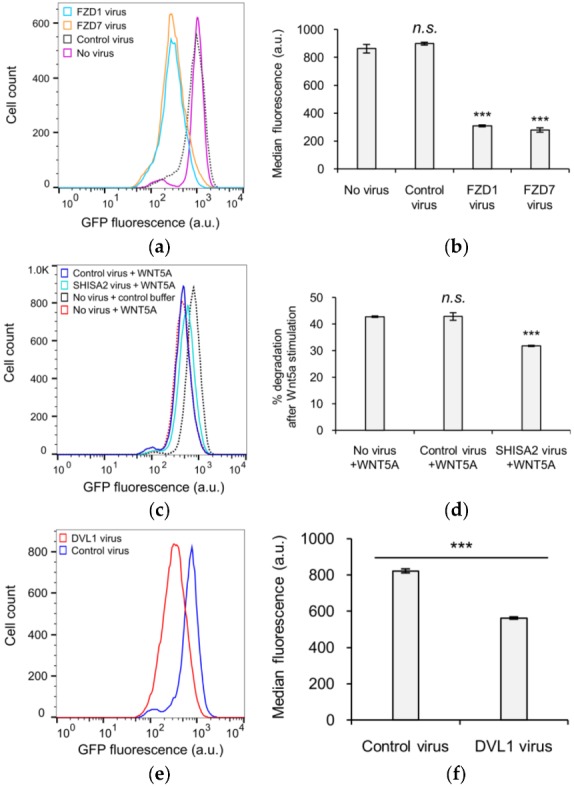
Functional characterization of KIF26B-C in the degradation reporter assay. (**a**) Flow cytometry histograms depicting the effect of ectopic FZD1 and FZD7 expression on GFP-KIF26B-C degradation (a.u. = arbitrary units); (**b**) Quantification of (**a**) (a.u. = arbitrary units). Median fluorescence is used for quantification because the experiment involved direct comparison of different cell lines, unlike the experiments presented in previous figures where % degradation was used to specifically express the effects of WNT5A stimulation within the same cell lines. Error bars represent ± SEM calculated from three independent replicates of each experimental condition. Statistical analysis was performed with unpaired *t*-test (*n.s.* = not significant; *** *p* value < 0.001 vs. no virus); (**c**) Flow cytometry histograms depicting the effect of ectopic SHISA2 expression on GFP-KIF26B-C degradation (a.u. = arbitrary units). Control buffer traces for “Control virus” and “SHISA2 virus” lines are similar to that of the “No virus” and thus not shown for visual clarity. However, data from the respective buffer control experiment for each virus-infected line were used for the quantification shown in (**d**); (**d**) Quantification of (**c**). Error bars represent ± SEM calculated from three independent replicates of each experimental condition. Statistical analysis was performed with unpaired *t*-test (*n.s.* = not significant; *** *p* value < 0.001 vs. no virus, +WNT5A); (**e**) Flow cytometry histograms depicting the effect of ectopic DVL1 expression on GFP-KIF26B-C degradation (a.u. = arbitrary units); (**f**) Quantification of (**e**) (a.u. = arbitrary units). Error bars represent ± SEM calculated from three independent replicates of each experimental condition. Statistical analysis was performed with unpaired *t*-test (*** *p* value < 0.001 vs. control virus).

We next investigated the biochemical mechanism(s) by which the WNT5A signal received at the cell surface is transmitted to the WRD domain within KIF26B-C to promote its degradation. In addition to the well-established noncanonical WNT5A receptors ROR1 and ROR2, the Frizzled (FZD) family of receptors and the Dishevelled (DVL) family of cytoplasmic scaffolding proteins have also been implicated in WNT5A signaling to KIF26B degradation [8,20,21,22]. In particular, our previous work showed that, like WNT5A, overexpression of FZDs in NIH/3T3 cells induces robust degradation of full-length KIF26B, whereas overexpression of SHISA2, an antagonist of FZDs, partially blocks the ability of WNT5A to induce KIF26B degradation [8]. In addition, overexpression of DVL1 is also sufficient to induce KIF26B degradation [8]. To verify that the GFP-KIF26B-C reporter is as functionally competent as full-length KIF26B in receiving the WNT5A signal, we tested and found that overexpression of FZD1, FZD7, SHISA2 and DVL1 all had the same effects on GFP-KIF26B-C degradation as previously seen on the full-length GFP-KIF26B reporter (Figure 3a–f). We therefore conclude that the biochemical nature of the WNT5A signal that regulates KIF26B-C degradation is equivalent to that for full-length KIF26B, and that KIF26B-C can itself be used as a WNT5A-dependent degradation reporter in biochemical and cell biological assays.

**Figure 4:**
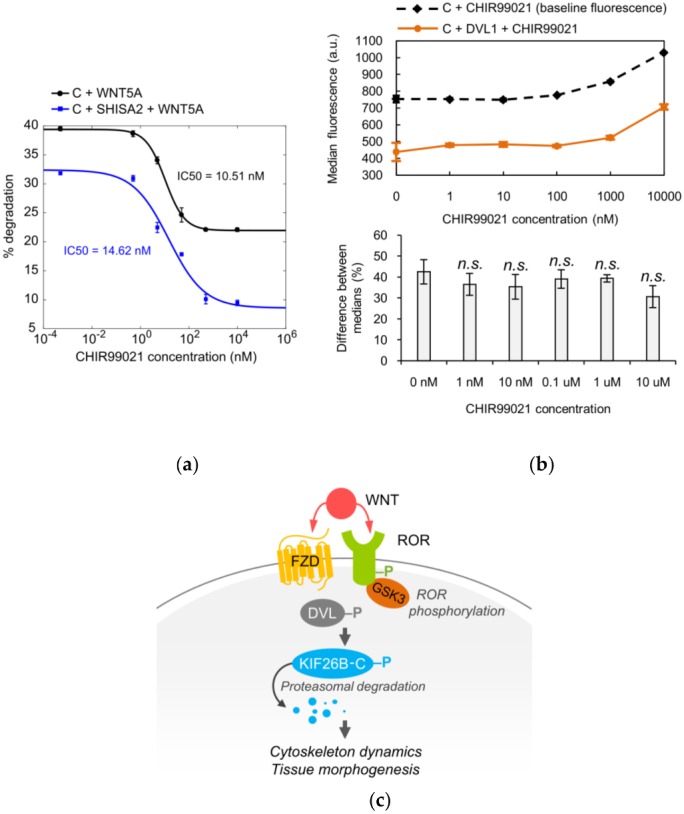
Glycogen synthase kinase 3 (GSK3) is part of the WNT5A signaling cascade that controls KIF26B-C degradation. (**a**) Dose-response curves showing WNT5A-induced GFP-KIF26B-C (C) degradation as a function of CHIR99021 (GSK3 inhibitor) concentration, without or with SHISA2 expression in the reporter cells. Error bars represent ± SEM calculated from independent replicates (*n* = 3 for each experimental condition); (**b**) **Top**: Median fluorescence vs. CHIR99021 concentration for GFP-KIF26B-C (baseline fluorescence) and GFP-KIF26B-C fluorescence resulting from DVL1-induced degradation (a.u. = arbitrary units). **Bottom**: quantification of the percent difference between DVL1-induced fluorescence and baseline medians for each concentration. Error bars represent ± SEM calculated from independent replicates (*n* = 6 for each experimental condition). For the quantification, statistical analysis was performed with unpaired *t*-test (*n.s.* = not significant vs. 0 nM); (**c**) Model of the role of GSK3 in FZD-ROR receptor dynamics.

To demonstrate the utility of KIF26B-C as a biochemical tool for dissecting the WNT5A-KIF26B signaling cascade, we tested several candidate molecules, focusing on the role of kinases. Glycogen synthase kinase 3 (GSK3), casein kinase (CK) and cyclin-dependent kinase (CDK) were previously implicated in WNT5A signaling or KIF26B regulation, but whether these kinases are specifically involved in WNT5A-dependent degradation of KIF26B has not been tested [23,24,25,26]. To investigate the role of these candidate kinases, we applied pharmacological inhibitors of these kinases to the GFP-KIF26B-C reporter cells and assessed the effects of these treatments on the ability of WNT5A to induce reporter degradation. We found that the GSK3 inhibitor CHIR99021 blocked WNT5A-induced GFP-KIF26B-C degradation with a calculated IC50 of 10.51 nM, within the range of the drug’s known on-target IC50 (Figure 4a) [27]. By contrast, in our assay system, the CK inhibitor D4476 and the CDK inhibitor Roscovitine did not block WNT5A-induced GFP-KIF26B-C degradation within the expected effective dose-range of these inhibitors [28]. These results indicate that GSK3 is a functional component of the WNT5A signaling cascade that controls KIF26B-C degradation. Interestingly, we noticed that even at saturating concentrations of CHIR99021, the inhibition of KIF26B-C degradation is partial (~50%; Figure 4a). Given that SHISA2 inhibition of KIF26B-C degradation is also partial (Figure 3d) and that ROR receptor activation was previously shown to involve GSK3-dependent phosphorylation [23,24], we hypothesized that the WNT5A signal can enter the cell through both FZD and ROR receptors, each of which is sensitive to inhibition by SHISA2 overexpression or the GSK3 inhibitor, respectively. This model predicts that simultaneous inhibition of FZD and GSK3 should more fully attenuate the ability of WNT5A to induce GFP-KIF26B-C degradation. To test this hypothesis, we determined the dose-response relationship between CHIR99021 and the inhibition of GFP-KIF26B-C degradation in cells that also expressed SHISA2 (Figure 4a). As predicted, when the FZD receptors are inhibited by SHISA2, CHIR99021 can more completely inhibit WNT5A-induced GFP-KIF26B-C degradation, as compared to CHIR99021 treatment in cells expressing GFP-KIF26B-C alone without SHISA2 (Figure 4a). This result supports the hypothesis that both ROR and FZD receptors participate in WNT5A-dependent degradation of KIF26B. Furthermore, we tested CHIR99021 on the GFP-KIF26B-C reporter cells that overexpressed DVL1 to determine if GSK3 is required for DVL-induced KIF26B degradation. Because DVL1 constitutively induces degradation of the reporter, we compared the median fluorescence resulting from DVL1-induced degradation of GFP-KIF26B-C to the baseline median fluorescence of GFP-KIF26B-C in the control reporter cells over a wide range of CHIR99021 concentrations (Figure 4b, top). We found that the GSK3 inhibitor did not significantly block DVL1-induced GFP-KIF26B-C degradation over the entire dose range (Figure 4b, bottom). We did notice that at very high doses (1000 nM and 10,000 nM), CHIR99021 treatment led to a slight increase in the expression of the reporter in both the control and DVL1-overexpressing cells; however, this global effect on reporter expression did not significantly alter the relative extent by which DVL1 downregulates the reporter signal (Figure 4b). Taken together, the observations that GSK3 inhibition can block WNT5A-induced, but not DVL1-induced, GFP-KIF26B-C degradation support the model in which GSK3 acts in the pathway upstream of DVL, likely at the level of ROR receptors (Figure 4c).

### 3.4. KIF26B-C as a Molecular Tool for Profiling Noncanonical WNT5A Signaling in Somatic and Stem Cells 

**Figure 5:**
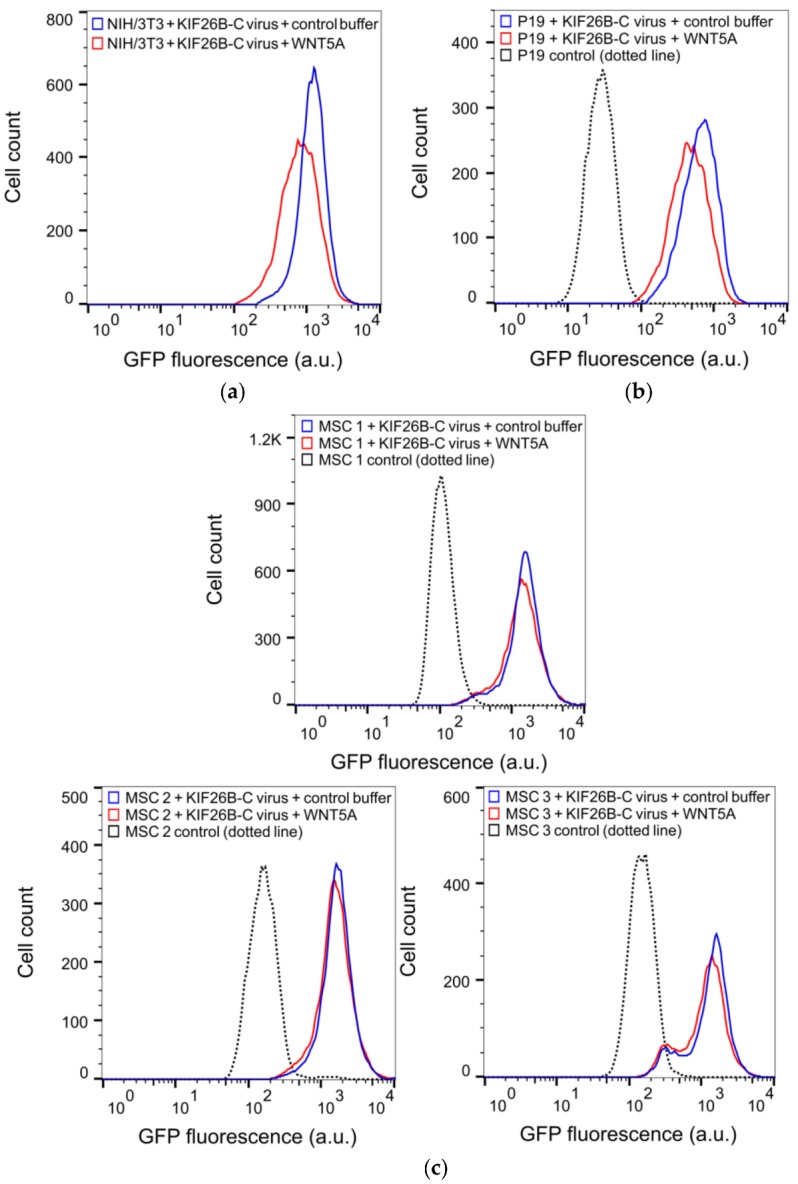

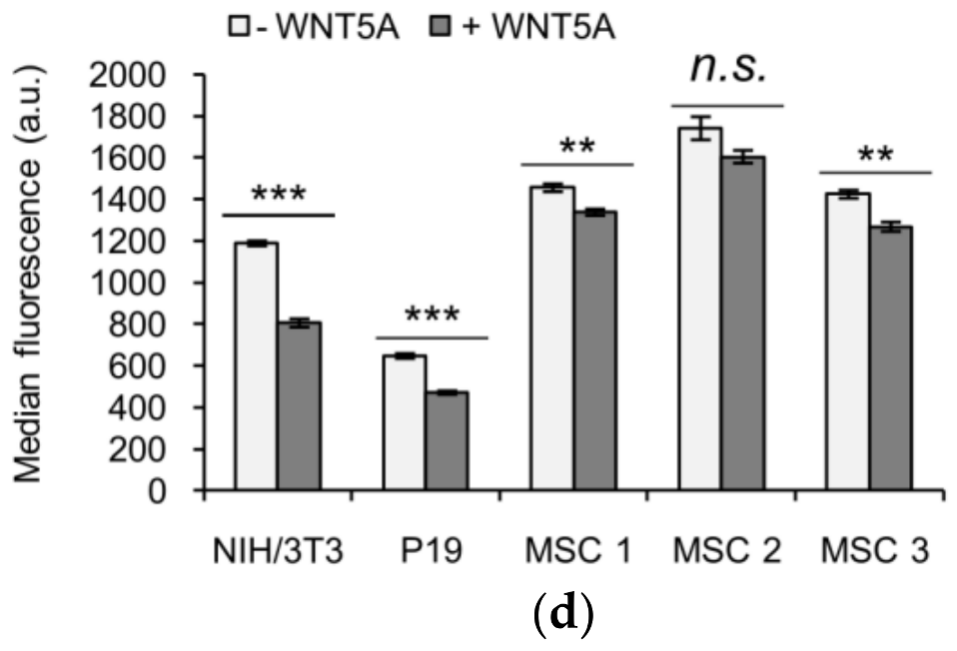
KIF26B-C as a functional reporter for profiling WNT5A signaling activity in somatic and stem cell lines. (**a**) Flow cytometry histograms depicting the degradation response of the lentiviral GFP-KIF26B-C reporter in NIH/3T3 cells after WNT5A stimulation (a.u. = arbitrary units); (**b**) Flow cytometry histograms depicting the degradation response of the lentiviral GFP-KIF26B-C reporter in P19 cells after WNT5A stimulation. Dotted trace indicates the autofluorescence of the control, uninfected cells (a.u. = arbitrary units); (**c**) Flow cytometry histograms depicting the degradation response of the lentiviral GFP-KIF26B-C reporter in three independent lines of primary human MSCs after WNT5A stimulation. Dotted trace indicates the autofluorescence of the control, uninfected cells (a.u. = arbitrary units); (**d**) Quantification of (**a**–**c**) (a.u. = arbitrary units). Error bars represent ± SEM calculated from independent replicates (*n* = 3 for NIH/3T3, P19; *n* = 4 for MSC 1, 2 and 3). Statistical analysis was performed with unpaired *t*-test (*n.s.* = not significant; *** *p* value < 0.001; ** *p* value < 0.01).

Our finding that KIF26B-C can fully recapitulate WNT5A-dependent KIF26B degradation as seen in the full-length KIF26B protein suggests that this assay can be used as a more compact and versatile reporter to quantify WNT5A-KIF26B signaling in live cells. This feature is important because so far in our study, we have exclusively used the Flp-In strategy to stably express the GFP-KIF26B reporters in an engineered, Flp-In-compatible NIH/3T3 line. However, the Flp-In system is currently not available for most cell types, include primary and stem cells. For these cells, the lentiviral expression system is the method of choice for introducing the GFP-KIF26B reporter constructs. The lentiviral expression system, however, has an insert size limit and the large size of the full-length *KIF26B* open reading frame complicates the use of this system. The substantially reduced size of KIF26B-C (about 1/5 the size of the full-length protein) thus makes it much easier to express via lentiviral transduction. In a proof-of-concept experiment, we used recombinant lentiviruses to deliver and express GFP-KIF26B-C in NIH/3T3 cells and tested the reporter function by assaying WNT5A-induced GFP-KIF26B-C degradation. As predicted, the reporter was efficiently expressed and properly regulated by WNT5A (Figure 5a,d). The extent of degradation was comparable to that seen in NIH/3T3 reporter cells generated via the Flp-In strategy. Thus, the lentiviral expression system can be used to express a functional GFP-KIF26B-C reporter in cells.

To further evaluate the utility of the lentivirus-based reporter system beyond NIH/3T3 cells, we tested this reporter system in two additional cell types, P19 embryonic carcinoma cells and primary human MSCs. P19 is a teratoma-derived line that exhibits several embryonic stem cell-like properties and have been used historically as a model to study the mechanisms of embryonic cell layer formation in vitro [29,30]. MSCs are multipotent stem cells capable of differentiating into a wide range of specialized cell types, including chondrocytes, osteocytes and adipocytes and thus have enormous therapeutic potentials in regenerative medicine [31,32]. Importantly, while noncanonical WNT5A-ROR signaling has been suggested to function in MSCs [33,34,35,36,37,38,39], direct evidence of pathway activation has been difficult to obtain due to the lack of a live cell reporter system. The lentiviral GFP-KIF26B-C reporter system offers the opportunity to functionally profile these cells based on their WNT5A signaling characteristics. We therefore used recombinant lentiviruses to express the GFP-KIF26B-C reporter in P19 cells and in primary MSC lines derived from three independent donors, stimulated these cells with WNT5A and quantified the extents by which GFP-KIF26B-C is degraded. We found that while P19 cells exhibited substantial GFP-KIF26B-C degradation activity, the MSC lines exhibited only minor to no degradation activity (Figure 5b–d). Together, these experiments demonstrate that the GFP-KIF26B-C reporter can be efficiently expressed in stem and stem-like cells and used to functionally profile WNT5A-KIF26B signaling activity in these cells. Given the broad tropism of recombinant lentiviruses, the GFP-KIF26B-C reporter system described here should be applicable in a wide spectrum of other mammalian cell types. 

## 4. Discussion

In this study, we determined the protein domain (WRD domain) required for WNT5A-dependent degradation of KIF26B. To the best of our knowledge, this is the first protein domain known to specifically mediate WNT5A-dependent protein degradation. The physiological importance of this domain is further emphasized by our finding that a recently reported human patient mutation located within this domain diminishes the ability of WNT5A to induce KIF26B degradation. As regulated degradation has emerged as a common strategy for noncanonical WNT signaling to control downstream effector function [5,6,7,8], the identification and characterization of the degradation domain of KIF26B represents a crucial step in understanding the mechanisms that underlie this WNT-dependent regulation. Defining the molecular and structural bases by which WRD integrates the WNT5A signal to promote KIF26B degradation represents an important future direction.

It is interesting to note that Terabayashi and colleagues previously reported that the C-terminus of KIF26B mediates CDK-induced degradation of KIF26B [26]. In addition, the authors demonstrated that CDK-dependent phosphorylation of two specific amino acid residues in KIF26B, Thr-1859 and Ser-1962, promotes the recruitment of the E3 ubiquitin ligase Nedd4 to KIF26B [26]. Collectively, this study and our present study support the crucial role of the KIF26B C-terminus in determining the stability of the KIF26B protein. However, we have tested and found that neither treatment with the CDK inhibitor Roscovitine nor substitutions of Thr-1859 and Ser-1962 with alanines significantly affected WNT5A-dependent KIF26B degradation [28]. Thus, the molecular mechanisms that underlie CDK- versus WNT5A-dependent KIF26B degradation appear to be distinct. It is possible that the C-terminus of KIF26B functions as a signaling node to integrate multiple signals within the cell to promote KIF26B degradation.

Our work also identified a specific role of GSK3 in transmitting the WNT5A signal to promote KIF26B degradation. Using biochemical epistasis analyses, we provide functional evidence that GSK3 acts in the pathway at a step upstream of DVL and likely at the level of the ROR receptors. As misregulations of WNT5A, ROR1, ROR2 and KIF26B expression/function have all been implicated in the pathogenesis of human diseases including cancer and congenital disorders [11,40,41,42,43,44,45,46,47], delineating the precise mechanism(s) by which pathway components, such as GSK3, modulate flux through the pathway should facilitate the development of therapeutic agents that target the pathway. Importantly, the WRD-based KIF26B degradation reporter assay described in this study, which reads out noncanonical WNT5A signaling activity in live cells, can serve as a powerful platform for screening of additional small molecule and/or protein modulators of the pathway.

In addition to its critical role in embryonic development and cancer progression, noncanonical WNT signaling has also been shown to participate in various aspects of stem cell biology. For example, WNT5A activity is required for axial elongation of P19 cell-derived organoids in vitro [48], and in MSCs, high noncanonical WNT signaling activity promotes osteogenic [49,50], chondrogenic [51] and neurogenic differentiation [52]. Progress in the field, however, has been hampered by the lack of a live-cell assay for directly measuring WNT5A signaling responses in these cells. In this study, we demonstrate that the WRD-based KIF26B degradation reporter assay can be efficiently expressed in P19 and primary human MSCs and used to profile WNT5A-KIF26B signaling activity. Interestingly, we found that while the KIF26B degradation response is relatively high in P19 cells, it is only marginally detected in MSCs. It is possible that only a small population of the cells in our MSC cultures is intrinsically competent to undergo WNT5A-KIF26B signaling, or that specific contexts such as the differentiation status of the cells or the cell culture conditions/substrates influence the activity of the pathway in these cells. In addition, since MSCs express relatively high levels of endogenous WNT5A [53], an autocrine signaling loop may exist, thereby inducing basal degradation of KIF26B even in the absence of exogenously added WNT5A. The WRD-based KIF26B degradation reporter assay can be used in future studies to distinguish these possibilities and more generally, to dissect the role of noncanonical WNT signaling in various types of somatic and stems cells.

## Appendix A

**Table A1:** List of primers used to generate mutant variants of *KIF26B and KIF26A*.

Mutant Name	Forward/Reverse	Primer Sequence (5′→3′)
KIF26B-Delta N	F	gatcggccggcctaccatgcggaagaagcagatcacc
KIF26B-Delta N	R	gatcggcgcgccttatcggcgcctggaggtgatgtc
KIF26B-Delta M	F	gatcggccggcctaccatgaagaccaagtacacatcaa
KIF26B-Delta M	R	gatcggcgcgccttatcggcgcctggaggtgatgtc
KIF26B-Delta C	F	gatcggccggcctaccatgaattcggtagccggaaataaag
KIF26B-Delta C	R	gatcggcgcgccttacttgctcactgcagagatctt
KIF26B-C	F	gatcggccggcctaccatgtctaagatctctgcagtga
KIF26B-C	R	gatcggcgcgccttatcggcgcctggaggtgatgtc
KIF26B-C1	F	gacttgaattcaggccggcctaccatgtctaagatctctg
KIF26B-C1	R	tatagttctagaggcgcgccttatcggcgcctggaggtgatgtc
KIF26B-C2	F	gacttgaattcaggccggcctaccatgtctaagatctctg
KIF26B-C2	R	tatagttctagaggcgcgccttagccgtccacccagcggac
KIF26B-C3	F	gacttgaattcaggccggcctaccaagcccgcagccgcccac
KIF26B-C3	R	tatagttctagaggcgcgccttatcggcgcctggaggtgatgtc
KIF26B-C4	F	gacttgaattcaggccggcctacccgctgggtggacggc
KIF26B-C4	R	tatagttctagaggcgcgccttatcggcgcctggaggtgatgtc
KIF26B-C5	F	gccggaaccaattcagtcgacaccatgccctcgccctacagcaag
KIF26B-C5	R	ccttgctcaccatggttgtggcgcctcggcgcctggaggtgatg
KIF26B-C6	F	gccggaaccaattcagtcgacaccatgacccctccgaggaagccg
KIF26B-C6	R	ccttgctcaccatggttgtggcgcctcggcgcctggaggtgatg
KIF26B-C7	F	gccggaaccaattcagtcgacaccatgagcagcgggcacggtagt
KIF26B-C7	R	ccttgctcaccatggttgtggcgcctcggcgcctggaggtgatg
KIF26B-C8	F	gccggaaccaattcagtcgacaccatgctaccacctgccatggg
KIF26B-C8	R	ccttgctcaccatggttgtggcgcctcggcgcctggaggtgatg
KIF26B-C9	F	gacttgaattcaggccggcctaccaagcccgcagccgcccac
KIF26B-C9	R	gatcggcgcgccttacaagcgctctgtcacacc
KIF26B-C10	F	gacttgaattcaggccggcctaccaagcccgcagccgcccac
KIF26B-C10	R	gatcggcgcgccttacaggtactgcttggtcgc
KIF26B-C11	F	gacttgaattcaggccggcctaccaagcccgcagccgcccac
KIF26B-C11	R	gatcggcgcgccttatcgacgtcgctgcaggcg
KIF26B-C (D1904N)	F	ggctacgagagcatgatgagaaacagcgaggccaccggcagtg
KIF26B-C (D1904N)	R	cactgccggtggcctcgctgtttctcatcatgctctcgtagcc
KIF26A-C	F	gatcggccggcctaccatgagcccagccaagggtgttggag
KIF26A-C	R	gatcggcgcgcctcaaacatccacctcttgtggccc
